# Establishment and Validation of a Novel MRI Radiomics Feature-Based Prognostic Model to Predict Distant Metastasis in Endemic Nasopharyngeal Carcinoma

**DOI:** 10.3389/fonc.2022.794975

**Published:** 2022-03-23

**Authors:** Hao-Jiang Li, Li-Zhi Liu, Ying Huang, Ya-Bin Jin, Xiang-Ping Chen, Wei Luo, Jian-Chun Su, Kai Chen, Jing Zhang, Guo-Yi Zhang

**Affiliations:** ^1^ Department of Radiology, State Key Laboratory of Oncology in Southern China, Collaborative Innovation Center for Cancer Medicine, Guangdong Key Laboratory of Nasopharyngeal Carcinoma Diagnosis and Therapy, Sun Yat-Sen University Cancer Center, Guangzhou, China; ^2^ Department of Radiation Oncology, State Key Laboratory of Oncology in Southern China, Collaborative Innovation Center for Cancer Medicine, Guangdong Key Laboratory of Nasopharyngeal Carcinoma Diagnosis and Therapy, Sun Yat-Sen University Cancer Center, Guangzhou, China; ^3^ Clinical Research Institute, Foshan Academy of Medical Sciences, Sun Yat-Sen University Foshan Hospital and The First People’s Hospital of Foshan, Foshan, China; ^4^ Department of Radiation Oncology, Foshan Academy of Medical Sciences, Sun Yat-Sen University Foshan Hospital and The First People’s Hospital of Foshan, Foshan, China

**Keywords:** MRI, radiomics, nasopharyngeal carcinoma, prognosis, predictive model

## Abstract

**Purpose:**

We aimed to establish a prognostic model based on magnetic resonance imaging (MRI) radiomics features for individual distant metastasis risk prediction in patients with nasopharyngeal carcinoma (NPC).

**Methods:**

Regression analysis was applied to select radiomics features from T1-weighted (T1-w), contrast-enhanced T1-weighted (T1C-w), and T2-weighted (T2-w) MRI scans. All prognostic models were established using a primary cohort of 518 patients with NPC. The prognostic ability of the radiomics, clinical (based on clinical factors), and merged prognostic models (integrating clinical factors with radiomics) were identified using a concordance index (C-index). Models were tested using a validation cohort of 260 NPC patients. Distant metastasis-free survival (DMFS) were calculated by using the Kaplan-Meier method and compared by using the log-rank test.

**Results:**

In the primary cohort, seven radiomics prognostic models showed similar discrimination ability for DMFS to the clinical prognostic model (P=0.070-0.708), while seven merged prognostic models displayed better discrimination ability than the clinical prognostic model or corresponding radiomics prognostic models (all P<0.001). In the validation cohort, the C-indices of seven radiomics prognostic models (0.645-0.722) for DMFS prediction were higher than in the clinical prognostic model (0.552) (P=0.016 or <0.001) or in corresponding merged prognostic models (0.605-0.678) (P=0.297 to 0.857), with T1+T1C prognostic model (based on Radscore combinations of T1 and T1C Radiomics models) showing the highest C-index (0.722). In the decision curve analysis of the validation cohort for all prognostic models, the T1+T1C prognostic model displayed the best performance.

**Conclusions:**

Radiomics models, especially the T1+T1C prognostic model, provided better prognostic ability for DMFS in patients with NPC.

## Introduction

Nasopharyngeal carcinoma (NPC) is endemic to southern China, southeastern Asia, and northern Africa, where the peak incidence rate is 20–50 cases per 100 000 individuals (1, 2). Advances in NPC diagnosis and treatment using MRI, intensity-modulated radiotherapy, and combined chemotherapy, has significantly improved locoregional control, and distant metastasis is regarded as the main cause of treatment failure (3–5). In a meta-analysis that reviewed 13304 participants, intensity-modulated radiotherapy was associated with better 5-year locoregional control (odds ratio 2.08; 95% confidence interval [CI]=1.82–2.37), while there were no significant differences seen in distant metastasis-free survival (DMFS) compared with conventional radiotherapy (4). Although the current 5-year overall survival rate of NPC patients reach 80-88%, 15–25% of patients develop distant metastasis after treatment, especially in advanced NPC (5, 6), so to identify high-risk patients to guide optimal treatment decisions is vital.

Currently, the TNM staging system is widely used to predict prognosis and establish treatment strategies among patients with NPC. However, patients at the same clinical stage receiving similar treatments often have different outcomes (7). Among NPC patients with distant metastasis after treatment, nearly 80% are stage III or IV (8), when it is difficult to identify high-risk patients. The presence of plasma Epstein-Barr virus (EBV) DNA influences the disease process in NPC patients and is regarded as a potential biomarker with clinical applications (9). Other risk factors have been associated with prognosis in patients with NPC, including age, sex, smoking status, hepatitis B surface antigen, serum lactate dehydrogenase, high-sensitivity C-reactive protein, and microRNA or mRNA; however, results are inconsistent (10–15). Therefore, the prognostic value of these markers needs confirmation.

Radiomics focuses on extracting quantitative features from medical images to find a possible association with tumor phenotypic characteristics (16). Radiomic features were found to be associated with gene and/or protein expression profiles, treatment response, and clinical outcomes in different cancer types (16, 17). Pretreatment MRI radiomics or MRI-based radiomics nomograms were proposed to predict locoregional recurrence, distant metastasis, and early response to induction chemotherapy in endemic NPC (18–29). However, there is no effective radiomics-based model to distinguish different risks levels for distant metastasis among NPC patients.

We conducted a study of multiparametric MRI radiomics features to identify and validate an MRI-based radiomics model discriminating distant metastasis in patients with NPC. Moreover, we compared the accuracy of a radiomics prognostic model (based on selected radiomics features), a clinical prognostic model (based on clinical risk factors), and a prognostic model merging the two, for discriminating distant metastasis in NPC patients.

## Methods

### Participants

This retrospective study was approved by the Clinical Research Ethics Committee of the Sun Yat-Sen University Cancer Center (SYSUCC); pre-treatment written informed consent was obtained from all patients or their next of kin. The authenticity of the study was validated by uploading the fully raw data onto the Research Data Deposit (RDD) public platform (http://www.researchdata.org.cn), with the approval RDD number as RDDA 2021135093. There were 903 patients newly diagnosed with untreated and non-metastatic NPC initially enrolled between January 2010 and November 2012; 125 patients were excluded from analysis (see **Supplementary Materials**). The remaining 778 NPC patients were randomly assigned in a 2:1 proportion to the primary (n=518) or validation cohort (n=260).

All patients underwent pretreatment evaluation, including clinical examinations of the head and neck region, fiber optic nasopharyngoscopy, neck and nasopharynx MRI, chest radiography, abdominal sonography, and whole-body bone scan, and staged according to the eighth American Joint Committee on Cancer TNM staging manual (30). Pre-treatment blood samples were collected to determine blood type and the presence or levels of EBV DNA, hepatitis B surface antigen, lactate dehydrogenase, high-sensitivity C-reactive protein, platelets, and leucocytes. A quantitative polymerase chain reaction method was used for detecting plasma EBV DNA (31). Clinical patient characteristics are listed in **Table 1**.

**Table 1:** Patient demographic characteristics in the primary and validation cohorts.

Characteristic	Primary cohort (*N*=518)	Validation cohort (*N*=260)	*P*
Age (years)			0.944
Median (IQR)	44 (38–53)	46 (38–52)	
Gender			0.389
Male	371 (71.6%)	194 (74.6%)	
Female	147 (28.4%)	66 (25.4%)	
WHO pathologic classification			0.253
Type I/II	27 (5.2%)	19 (7.3%)	
Type III	491 (94.8%)	241 (92.7%)	
T classification			0.389
T1	140 (27%)	61 (23.5%)	
T2	60 (11.6%)	37 (14.2%)	
T3	186 (35.9%)	103 (39.6%)	
T4	132 (25.5%)	59 (22.7%)	
N classification			0.425
N0	128 (24.7%)	53 (20.4%)	
N1	282 (54.4%)	151 (58.1%)	
N2	69 (13.3%)	40 (15.4%)	
N3	39 (7.5%)	16 (6.2%)	
AJCC clinical stage (2010)			0.181
I	53 (10.2%)	19 (7.3%)	
II	117 (22.6%)	57 (21.9%)	
III	185 (35.7%)	112 (43.1%)	
IVa	163 (31.5%)	72 (27.7%)	
Treatment regimen			0.490
RT alone	71 (13.7%)	34 (13.1%)	
CCRT	201 (38.8%)	91 (35%)	
IC + CCRT	246 (47.5%)	135 (51.9%)	
EBV DNA (10^3^ copies/ml)*			0.262
<1	232 (44.8%)	125 (48.1%)	
<10	117 (22.6%)	67 (25.8%)	
<100	129 (24.9%)	49 (18.8%)	
≥100	40 (7.7%)	19 (7.3%)	
Blood type			0.145
A	139 (26.8%)	56 (21.5%)	
B	130 (25.1%)	67 (25.8%)	
AB	17 (3.3%)	16 (6.2%)	
o	232 (44.8%)	121 (46.5%)	
HBsAg			0.620
Negative	427 (82.4%)	210 (80.8%)	
Positive	91 (17.6%)	50 (19.2%)	
LDH (U/L)*			0.142
<245	492 (95%)	240 (92.3%)	
≥245	26 (5%)	20 (7.7%)	
hs-CRP (g/ml)*			0.186
<1	196 (37.8%)	101 (38.8%)	
1–3	166 (32%)	96 (36.9%)	
≥3	156 (30.1%)	63 (24.2%)	
Platelet counts (10^9^/L)*			0.459
<100	4 (0.8%)	2 (0.8%)	
100–300	436 (84.2%)	227 (87.3%)	
≥300	78 (15.1%)	31 (11.9%)	
Leucocyte counts (10^9^/L)*			0.347
<4	9 (1.7%)	9 (3.5%)	
4–10	457 (88.2%)	225 (86.5%)	
≥10	52 (10%)	26 (10%)	
Follow-up time (months)			0.886
Median (min, max)	84.6 (3.3–104.1)	84.4 (6.9–103.9)	

IQR, interquartile range; WHO, World Health Organization; Type I, keratinizing; Type II, non-keratinizing differentiated; Type III, non-keratinizing undifferentiated; T, tumor; N, node; AJCC, American Joint Committee on Cancer; RT, radiotherapy; CCRT, concurrent chemoradiotherapy; IC, induction chemotherapy; EBV DNA, Plasma Epstein–Barr virus DNA; HBsAg, hepatitis B surface antigen; LDH, serum lactate dehydrogenase levels; hs-CRP, high-sensitivity C-reactive protein; min, minimum; max, maximum. *Results before treatment.

All patients were treated according to the SYSUCC guidelines for NPC patients, which recommend that stage I patients have to receive radiotherapy alone, stage II radiotherapy alone, or concomitant chemoradiotherapy, and stage III–IVa concomitant chemoradiotherapy with or without induction chemotherapy. All patients received definitive intensity-modulated radiotherapy with 6-MV photons during the entire radiotherapy course. Concurrent chemotherapy, induction chemotherapy, and intensity-modulated radiotherapy data are presented in the **Supplementary Material**.

Complete follow-up data at 3, 5, and 8 years were available for 97.8%, 96.4%, and 87.6% of patients, respectively. Follow-up visits occurred at least once every 3 months during the first 3 years and then once every 6 months after treatment. DMFS was calculated from the date of treatment initiation to that of distant metastasis at any site, death from any cause, or the date of the last follow-up. Distant metastases were assessed using two imaging methods, including chest radiography, abdominal sonography, bone scan, and CT or MRI; elevation of plasma EBV DNA was confirmed by pathological biopsy if necessary.

### MRI Acquisition/Segmentation and Radiomics Feature Extraction

All patients underwent MRI with a 1.5-T system (Signa CV/i; General Electric Healthcare, Chalfont St. Giles, United Kingdom) or 3.0 T system (Siemens Magnetom Tim Trio, Erlangen, Germany), employing the fast spin-echo technique. The scan region imaged ranged from the suprasellar cistern to the inferior margin of the sternal end of the clavicle using a head-and-neck combined coil. T1-weighted images (T1-w) in the axial, coronal, and sagittal planes, T2-weighted (T2-w) images in the axial plane, and contrast-enhanced T1-weighted (T1C-w) in the axial, coronal, and sagittal planes were acquired in all patients. Detailed information on MRI scans is shown in Supplementary Materials.

Axial T1-w, T2-w, and T1C-w Digital Imaging and Communications in Medicine (DICOM) images were retrieved from the picture archiving and communication system (PACS) and loaded into AnalyzePro (https://analyzedirect.com/analyzepro/) for semi-manual segmentation with normalization. One radiologist (Z. Guoyi) with over 10 years of experience in head and neck cancers outlined the tumor contour regions of interest (ROI) on each MRI slice, and each segmentation was validated by a senior radiologist (L. Lizhi) with 20 years of experience in evaluating MRI scans of patients with NPC. Differences were resolved by consensus. Extractions of radiomics features were performed using the open-source PyRadiomics (http://www.radiomics.io/pyradiomics.html). We extracted 4527 radiomics features for each patient from axial T1-w, T2-w, and T1C-w axial images (1509 features from each unimodal MR image). Data from the radiomics features are shown in the **Supplementary Material**.

### Statistical Analysis Methods

The interclass correlation coefficient was used to assess the effect of variations in manual segmentation on radiomics feature values from 50 patients randomly extracted from the primary cohort. Features with high reproducibility (median ± standard deviation: interclass correlation coefficient >0.80) were retained for subsequent analysis. After deleting features that had a Pearson correlation coefficient ≥0.75, univariate analysis using the Cox regression model was employed as the first step of feature selection to assess the possible risk factor of DMFS. The least absolute shrinkage and selection operator method was the second feature-selection process for model building. **Supplementary Figures S1, S2** show the processes of radiomics feature selection and radiomics feature selection using the least absolute shrinkage and selection operator regression model, respectively.

Kaplan-Meier survival curves and log-rank tests were used to evaluate the time-event data. Clinical characteristics between the primary and validation cohorts were compared using the chi-square test for categorical variables and the Mann-Whitney U test for continuous variables. Harrell’s concordance indices (C-indices) were applied to evaluate the discriminating ability of each prognostic model. The establishment flowchart of the clinical prognostic model, radiomics prognostic models, and merged prognostic models is shown in **Supplementary Figure S3**.

Statistical analyses were performed using R software version 3.2.5. with the following R packages: the caret package for Pearson correlation analysis; the survival package and survminer for Kaplan-Meier survival curves; the glmnet package for least absolute shrinkage and selection operator Cox regression; the ggplot2 package for score plot; the rms package for calibration curves and nomograms; the Hmisc package for comparisons between models in terms of C-indices; the pheatmap and gplots packages for heatmaps. All statistical tests were two-tailed with *P*<0.05 indicating statistical significance.

## Results

### Participants

**Table 1** lists the clinical characteristics of patients with NPC. No significant differences were found in clinical characteristics between the primary and validation cohorts (*P*=0.142 to 0.944). During the follow-up period, 12.9% (67/518) and 13.1% (34/260) of patients developed distant metastases in the primary and validation cohorts, respectively. There was no significant difference between the two cohorts in the distant metastases rate (*P*=0.961). The median DMFS time was 84.3 months (range, 3.3–104.1) for the primary cohort, and 84.4 months (range, 6.6–103.9) for the validation cohort.

### Clinical Prognostic Model Building

Univariate analysis indicated that pre-treatment T stage, N stage, clinical stage, and plasma EBV DNA were associated with DMFS in patients with NPC (**Supplementary Table S1**). Multivariable analyses further identified that pre-treatment T stage, N stage, and plasma EBV DNA were independent predictors for DMFS (**Supplementary Table S2**). The clinical prognostic model for DMFS discriminating was based on these three independent factors. The C-index of this model for the primary cohort was 0.736 (95% CI=0.680–0.791), significantly higher than the C-indices for the validation cohort, 0.552 (95% CI=0.457– 0.647) (**Table 2**).

**Table 2:** C-index values of different prognostic models for DMFS prediction in the primary and validation cohorts.

Prognostic model		Degree of freedom	Primary cohort			Validation cohort		
			C-index (95% CI)	*P*†	P*	C-index (95% CI)	*P*†	P*
Clinical prognostic model		9	0.736 (0.68 0.791)	Reference		0.552 (0.457, 0.647)	Reference	
Radiomics prognostic model	T1	7	0.723 (0.666, 0.780)	0.181	reference	0.676 (0.588, 0.764)	<0.001	reference
	T2	5	0.715 (0.656, 0.774)	0.232	reference	0.645 (0.546, 0.744)	0.016	reference
	T1C	9	0.733 (0.676, 0.791)	0.708	reference	0.711 (0.615, 0.807)	<0.001	reference
	T1+2	2	0.771 (0.720, 0.823)	0.175	reference	0.679 (0.594, 0.763)	<0.001	reference
	T1+1C	2	0.757 (0.703, 0.81)	0.468	reference	0.722 (0.632, 0.811)	<0.001	reference
	T2+1C	2	0.763 (0.712, 0.813)	0.430	reference	0.697 (0.599, 0.795)	<0.001	reference
	T1+2+1C	3	0.784 (0.737, 0.831)	0.070	reference	0.711 (0.622, 0.799)	<0.001	reference
Merged prognostic model	MT1	10	0.784 (0.731, 0.837)	<0.001	<0.001	0.640 (0.558, 0.723)	0.057	0.511
	MT2	10	0.773 (0.719, 0.826)	<0.001	<0.001	0.605 (0.500, 0.710)	0.055	0.647
	MT1C	10	0.791 (0.741, 0.841)	<0.001	<0.001	0.661 (0.563, 0.759)	0.003	0.857
	MT1+2	11	0.801 (0.752, 0.850)	<0.001	<0.001	0.648 (0.560, 0.735)	0.004	0.322
	MT1+1C	11	0.812 (0.763, 0.860)	<0.001	<0.001	0.678 (0.589, 0.767)	<0.001	0.391
	MT2+1C	11	0.806 (0.758, 0.854)	<0.001	<0.001	0.653 (0.554, 0.753)	<0.001	0.600
	MT1+2+1C	12	0.818 (0.771, 0.865)	<0.001	<0.001	0.677 (0.587, 0.767)	<0.001	0.297
Remerged prognostic model	rMT1	7	0.780 (0.726, 0.834)	<0.001	<0.001	0.653 (0.570, 0.736)	<0.001	0.843
	rMT2	7	0.773 (0.719, 0.827)	<0.001	<0.001	0.612 (0.508, 0.716)	0.046	0.964
	rMT1C	7	0.789 (0.739, 0.839)	<0.001	<0.001	0.671 (0.576, 0.766)	<0.001	0.893
	rMT1+2	8	0.799 (0.749, 0.848)	<0.001	<0.001	0.661(0.573, 0.748)	<0.001	0.480
	rMT1+1C	8	0.809 (0.761, 0.857)	<0.001	<0.001	0.685 (0.597, 0.774)	<0.001	0.663
	rMT2+1C	8	0.804 (0.757, 0.851)	<0.001	<0.001	0.665 (0.566, 0.763)	<0.001	0.874
	rMT1+2+1C	9	0.815 (0.769, 0.862)	<0.001	<0.001	0.683 (0.592, 0.775)	<0.001	0.385

Note.**—**C-index = concordance index; DMFS = distant metastasis-free survival; CI = confidence interval. MT1, MT2, MT1C, MT1+T2, MT1+T1C, MT2+T1C and MT1+T2+T1C prognostic models were built, and they integrated clinical risk factors (T stage, N stage, and plasma EBV DNA) with the T1, T2, T1C, T1+T2, T1+T1C, T2+T1C and T1+T2+T1C radiomics prognostic models, respectively. rMT1, rMT2, rMT1C, rMT1+T2, rMT1+T1C, rMT2+T1C and rMT1+T2+T1C prognostic models were built based on N stage, plasma EBV DNA with the T1, T2, T1C, T1+T2, T1+T1C, T2+T1C and T1+T2+T1C radiomics prognostic models, respectively.

^†^P-values were calculated compared with the clinical prognostic model.

*P-values were calculated by comparing with the corresponding radiomics prognostic model. For example, P=.0511 was the result of the comparison between the MT1 model and the T1 model in the validation cohort.

### Radiomics Prognostic Model Building

**Supplementary Table S3** summarizes the features most strongly associated with DMFS based on analysis of the primary cohort. The T1, T1c, and T2 prognostic models for DMFS prediction were based on selected radiomics features derived from T1-w, T1C-w, and T2-w MRI scans, respectively (**Table 3**). The T1+T1C, T1+T2, T2+T1C, and T1+T2+T1C prognostic models were based on Radscore combinations of the T1 and T1C, T1 and T2, T2 and T1C, and T1, T2 and T1C prognostic models, respectively (**Supplementary Figure S3** and **Table 3**).

**Table 3:** Multivariable analysis of radiomic features for the primary cohort.

Prognostic model	Variable	DMFS		
		coefficient	HR (95% CI)	*P*
T1 radiomics prognostic model	T1_shape_Sphericity	-4.67	0.01 (1.78E-04, 0.49)	0.021
	T1_W_LHH__GLSZM_LGLZE	-1.47	0.23 (0.04, 1.37)	0.107
	T1_W_HHL__GLCM_IMC2	-6.33	1.78E-03 (5.51E-05, 5.76E-02)	< 0.001
	T1_W_HHH__GLCM_IMC2	4.93	138.93 (2.39, 8.09E3)	0.017
	T1_W_HLH__GLCM_IMC2	-3.41	0.03 (6.41E-04, 1.71)	0.090
	T1_log.sigma.3.0.mm.3D_NGTDM_Strength	1.36	3.91 (1.19, 12.89)	0.025
	T1_W_HHH__NGTDM_Contrast	-14.64	4.37E-07(3.20E-16, 595.36)	0.172
T2 radiomics prognostic model	T2_W_HLL__GLDM_LDHGLE	8.04E-05	1.00008 (1.000006, 1.000115)	0.034
	T2_log.sigma.5.0.mm.3D_FOS_Skewness	-0.45	0.64 (0.38, 1.06)	0.084
	T2_W_HHL__GLSZM_SALGLE	-23.50	6.25E-11 (6.92E-18, 5.64E-04)	0.004
	T2_logarithm_NGTDM_Coarseness	17.62	4.48E+07 (1.22E+02, 1.65E+13)	0.007
	T2_W_LLH__GLCM_IDMN	25.21	8.86E+10 (1.22E-01, 6.45E+22)	0.070
T1C radiomics prognostic model	T1C_W_HLL__GLCM_Correlation	6.59	7.30E+02 (6.08, 8.77E+04)	0.007
	T1C_W_LLH__GLSZM_SAHGLE	0.03	1.03 (1.01, 1.05)	0.010
	T1C_Gradient_GLCM_IMC1	9.33	1.13E+04 (0.40, 3.23E+08)	0.075
	T1C_Square_GLCM_Correlation	3.22	25.02 (1.95, 3.21E+02)	0.013
	T1C_Gradient_GLSZM_ZE	0.60	1.83 (0.93, 3.58)	0.079
	T1C_square_GLRLM_RE	-1.66	0.19 (0.06, 0.61)	0.005
	T1C_log.sigma.3.0.mm.3D_GLSZM_ZE	0.94	2.55 (1.07, 6.07)	0.035
	T1C_W_LHL__GLSZM_ZE	-1.05	0.35 (0.11, 1.06)	0.063
	T1C_gradient_GLSZM_GLN	0.04	1.04 (0.99, 1.08)	0.104
T1+1C radiomics prognostic model	T1 radscore*	0.59	1.80 (1.25, 2.59)	0.002
	T1C radscore*	0.75	2.12 (1.53, 2.94)	<0.001
T1+2+1C radiomics prognostic model	T1 radscore*	0.42	1.52 (1.04, 2.23)	0.031
	T2 radscore*	0.48	1.61 (1.14, 2.28)	0.007
	T1C radscore*	0.58	1.79 (1.26, 2.54)	0.001

CI, confidence interval; DMFS, distant metastasis free survival; HR, hazard ratio. Textural features should be decomposed into three-dimensional wavelet transform (8 decompositions), and the wavelet decompositions are labeled as W_LLL_, W_LLH_, W_LHL_, W_LHH_, W_HLH_, W_HHL_, W_HHL_, and W_HHH_. T1-w = T1-weighted; T2-w = T2-weighted; T1C-w = contrast-enhanced T1-weighted. GLSZM, Gray Level Size Zone Matrix; GLCM, Gray Level Co-occurrence Matrix; NGTDM, Neighbouring Gray Tone Difference Matrix; GLDM, Gray Level Dependence Matrix; FOS, First order statistics; GLRLM, Gray Level Run Length Matrix; LGLZE, Low Gray Level Zone Emphasis; IMC, Informational measure of correlation; JA, Joint Average; LDHGLE, Large Dependence High Gray Level Emphasis; SALGLE, Small Area Low Gray Level Emphasis; IDMN, Inverse Difference Moment Normalized; SAHGLE, Small Area High Gray Level Emphasis; ZE, Zone Entropy; RE, Run Entropy; GLN, Gray Level Non-Uniformity.

*radscore from nomogram.

As shown in **Table 2**, in the primary cohort, seven radiomics prognostic models showed similar DMFS discriminating ability as that of the clinical prognostic model (*P*=0.070 to 0.708). While in the validation cohort, the C-indices for DMFS prediction by the seven models (0.645-0.722) were significantly higher than the C-index calculated using the clinical prognostic model (*P*=0.016 or <0.001). Intriguingly, in the seven radiomics models of the validation cohort, the T1+1C prognostic model showed the highest C-index (0.722, 95% CI=0.632-0.811) for DMFS discriminating, significantly superior to the C-indices of the T1 (0.676), T2 (0.645), and T1C (0.711) prognostic models (*P*=0.008, 0.043, and 0.048, respectively) and higher than those of the other three radiomics models, although without significant differences (**Supplementary Table S4**).

### Merged Prognostic Model Building

To identify whether integrating clinical factors with radiomics prognostic models could increase predictive accuracy, we built seven merged prognostic models (MT1, MT2, MT1C, MT1+T2, MT1+T1C, MT2+T1C, and MT1+T2+T1C) based on combinations of three clinical factors and the T1, T2, T1C, T1+T2, T1+T1C, T2+T1C, and T1+T2+T1C radiomics models, respectively (**Supplementary Figure S3**). In the primary cohort, merged prognostic models displayed better discriminating ability in DMFS than the corresponding radiomics prognostic models (all *P*<0.001). While in the validation cohort, the C-indices of seven merged prognostic models (0.605-0.678) for DMFS discriminating were all lower than those of the corresponding radiomics prognostic models; although, there were no significant differences (**Table 2**), indicating that adding the clinical factors to the radiomics models weakened their discriminating ability.

To confirm this finding, we removed the T stage from the merged prognostic models and constructed seven additional merged prognostic models (rMT1, rMT2, rMT1C, rMT1+T2, rMT1+T1C, rMT2+T1C, and rMT1+T2+T1C) based on the radiomics prognostic model, N stage, and EBV-DNA. The C-indices of these seven models (0.612-0.685) were slightly better than those of the corresponding merged prognostic models in the validation cohort (**Table 2**).

### Performance of the Clinical and T1+1C Prognostic Model

Based on the results above, T1+1C radiomics model was considered to provide the best prognostic ability for DMFS (having the highest C-index and Least degree of freedom). We further compared the discriminating ability between the clinical prognostic model and the T1+T1C prognostic model for DMFS from their nomograms, calibration curves, risk score distributions, and decision curve analysis.

**Figure 1:**
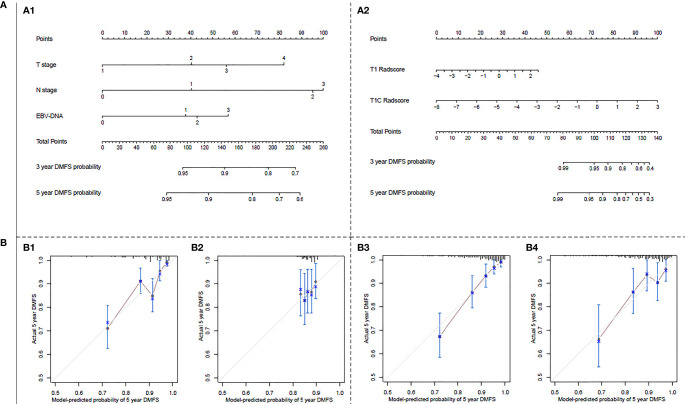
**(A)** Nomograms for 3- and 5-year distant metastasis-free survival (DMFS): (A1) for the clinical prognostic model, (A2) for the T1+T1C prognostic model. The nomogram allows the user to obtain the probability of 3- and 5-year DMFS corresponding to a patient combination of covariates. As an example, locate the patient T stage and draw a line straight upward to the “Points” axis to determine the score associated with that T stage. Repeat the process for each variable and sum of the scores achieved for each covariate; after that, locate this sum on the “Total Points” axis. Draw a line straight down to determine the likelihood of 3- or 5-year DMFS. **(B)** Calibration curves for predicting 5-year DMFS: (B1) in the primary cohort of the clinical prognostic model, (B2) in the validation cohort of the clinical prognostic model, (B3) in the primary cohort of T1+T1C prognostic model, (B4) in the validation cohort of T1+T1C prognostic model. The Y-axis shows observed survival estimated by the Kaplan–Meier method, and the X-axis shows predicted survival calculated using the prognostic model. The solid lines represent the ideal reference line for which predicted survival corresponds with actual survival.

Nomograms and calibration curves for the clinical and T1+T1C radiomics prognostic models are shown in **Figure 1**. The T1+T1C radiomics prognostic model predicted post-treatment metastasis risk for patients with a 3-year DMFS probability ≥40% or 5-year DMFS probability ≥30%, while the clinical prognostic model required a 3-year DMFS probability ≥70% or 5-year DMFS probability ≥60% to predicted metastasis risk (**Figures 1A1, A2**). Calibration curves in the probability of 5-year DMFS by a 1000-iteration bootstrap resampling experiment in the validation cohort also showed the T1+1C prognostic model had better discriminating ability than the clinical prognostic model. Observations showed better agreement with model predictions when the probability of 5-year DMFS was ≥68.9% in the T1+1C prognostic model, while it had to be ≥83.3% in the clinical prognostic model (**Figures 1B2, B4**).

**Figure 2:**
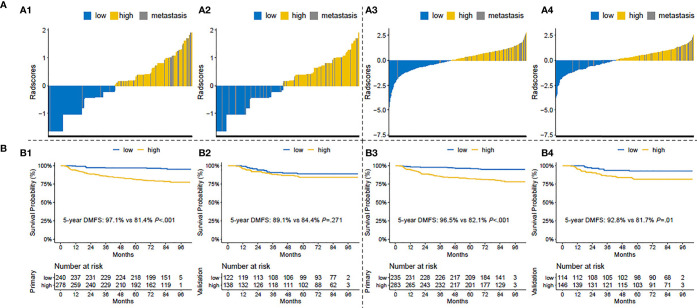
**(A)** Risk score distributions: (A1) in the primary cohort of the clinical prognostic model, (A2) in the validation cohort of the clinical prognostic model, (A3) in the primary cohort of the T1+T1C prognostic model, A4) in the validation cohort of the T1+T1C prognostic model. **(B)** Kaplan–Meier survival curves of distant metastasis-free survival (DMFS) in patients of the low- and high-risk groups: (B1) in the primary cohort of the clinical prognostic model, (B2) in the validation cohort of the clinical prognostic model, (B3) in the primary cohort of the T1+T1C prognostic model, (B4) in the validation cohort of the T1+T1C prognostic model.

We calculated the risk scores in the clinical and T1+T1C prognostic models for each patient and then classified patients into the low- and high-risk groups, with zero as risk score cutoff. The distributions of risk scores and 5-year DMFS in the low- and high-risk groups are shown in **Figure 2**. As shown, post-treatment metastatic patients were concentrated in the high score area, and the survival curve of the T1+T1C prognostic model showed good prognostic stratification for patients in the low- and high-risk groups in the validation cohort, while such trends were not observed in the validation cohort of the clinical prognostic model. When maximally selected rank statistics were used to generate the optimal risk score cutoff value for the clinical and T1+T1C prognostic models to divide patients into low- and high-risk groups, the results were identical to when zero was used as cutoff value (**Supplementary Figures S4, 5**).

**Figure 3:**
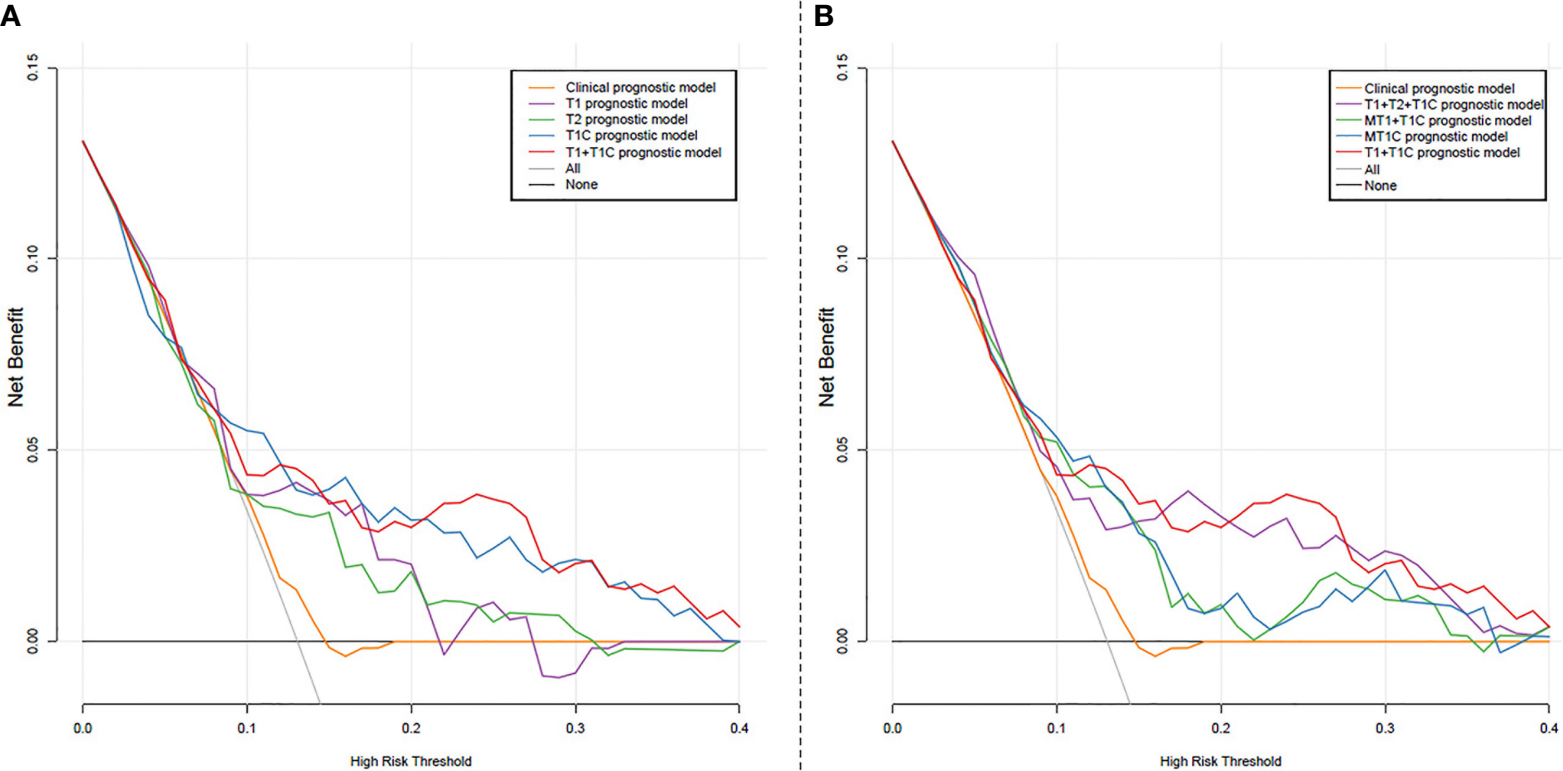
Decision curve analysis for the prognostic models: **(A)** Clinical prognostic model, and radiomics models T1, T2, T1C, T1+T1C; **(B)** Clinical prognostic model, radiomics models T1+T2+T1C, T1+T1C, MT1+T1C model, and rMT1C mode. The X-axis represents the probability of the 5-year distant metastasis-free survival ranging from 0 to 100%. The Y-axis shows the net benefit. The black line indicates that no distant metastasis occurred in all patients. The gray line represents the assumption that all patients developed distant metastasis.

The decision curve analysis of the validation cohort for the clinical prognostic model, radiomics models T1, T2, T1C, T1+T1C, T1+T2+T1C, MT1+T1C model, and rMT1C model is shown in **Figure 3** (other prognostic models are not listed). The T1+T1C prognostic model provided the best performance, while the clinical prognostic model showed no net benefit for patients with a 5-year DMFS probability ≥14.9%.

## Discussion

Our radiomics prognostic models, especially the T1+T1C prognostic model, had better discriminating ability than either the clinical prognostic model or corresponding merged prognostic models integrating clinical factors with selected radiomics features. All seven radiomics prognostic models could stratify patients into high- and low-risk groups based on significantly different 5-year DMFS rates. To our knowledge, this is the first study to clarify the role of multiple versus single MRI sequence radiomics, of radiomics features versus clinical factors, or the combination of clinical factors and radiomics features to predict prognosis with such a large sample size of nasopharyngeal carcinoma patients. Our findings could be useful for the evaluation of individual distant metastasis risk and the choice of a personalized therapeutic regimen in nasopharyngeal carcinoma patients.

The TNM staging system is routinely used in clinical practice to guide assessment of individual prognosis and treatment strategy decisions. However, an obvious shortcoming of the TNM staging system is that its basis on anatomical tumor extent does not reflect intra-tumor heterogeneity, which has pronounced effects on tumor diagnosis and prognosis. Plasma EBV DNA is a potential biomarker for NPC clinical management, but the lack of a global standardized testing methodology limits its widespread value (9). Since NPC is spatially and temporally heterogeneous (32), plasma EBV DNA does not completely characterize tumor heterogeneity. In this study, the C-index of the clinical prognostic model was 0.736 in the primary cohort, while it was significantly reduced to 0.552 in the validation cohort, indicating that clinical risk factors were inadequate for distinguishing between different metastasis risk groups and not a reliable method for predicting DMFS in NPC patients.

Radiomics transforms tumor images into detailed quantifications of tumor characteristics to find possible prognostic information for patients (16). Our study showed that radiomics features could be used as biomarkers for DMFS prediction in NPC patients without considering the TNM staging system or other clinical risk factors. There might be two reasons for this result. First, clinical risk factors could not distinguish between different metastasis risks; therefore, adding clinical factors into the radiomics prognostic models could not significantly improve the models’ prediction efficiency. Second, the selected radiomics features might contain prognostic information hidden in clinical factors, and overfitting occurrence might weaken their prediction performance when integrating clinical factors with radiomics prognostic models. Zhang et al. (21) showed that the C-index of a radiomics prognostic model using the TNM staging system or clinical factors (based on age, sex, and hemoglobin) was reduced by 0.009 or 0.013 in the validation cohort, respectively.

T1-w, T1C-w, and T2-w are three common methods used in MRI tumor diagnosis. In single-MRI prognostic models for DMFS, the T1C prognostic model had the highest discriminating ability in our study, followed by the T1 and T2, in both primary and validation cohorts. Differences in discriminating ability may be due to imaging capabilities. T1-w can detect tumor anatomical details; T2-w images are sensitive for detecting effusion or edema; T1C-w images may reflect tumor angiogenesis, closely related to tumor invasion and metastasis. For multiple-MRI prognostic models for DMFS, the T1+T1C prognostic model had the best performance in the validation cohort, with a higher C-index than the T1+T2, T2+T1C or T1+T2+T1C prognostic models. Among all prognostic models, the T1+T1C prognostic model also showed the best performance in the decision curve analysis of the validation cohort. Therefore, the radiomics features from T1-w and T1C-w MRI sequences better characterize metastatic biological behaviors than other combinations of the three MRI modalities. Except for one shape fature (sphericity), the other 15 features of the composition of T1+T1C radiomics model are textural features, which reflect tumor heterogeneity. This indicates a radiomic signature of some textural features can be reguarded as a potential prognostic biomark to discriminate different metastasis risk groups in NPC patients.

Our study had several limitations. First, the patients in the primary and validation cohorts were enrolled from the same institution, which may reduce the generalizability of our findings. Second, follow-up was incomplete for some patients, which may have led to distortions. Third, although PyRadiomics adheres for the most part to the Image Biomarker Standardization Initiative (IBSI), some small differences have been duly noted for it in terms of quantifying the grey level values, removing features associated with the ROI volum from the very beginning for example. Fourth, other MRI-based radiomics were not included, especially diffusion-weighted imaging and dynamic contrast-enhanced, significant for treatment monitoring and outcome prediction in different cancer types (33–35). Thus, the usefulness of diffusion-weighted imaging and dynamic contrast-enhanced MRI-based radiomics in NPC patients should be further explored.

In conclusion, a T1+T1C radiomics prognostic model could be a reliable approach for individual risk discriminating of distant metastasis in nasopharyngeal carcinoma patients. This model provided better prognostic performance than other radiomics prognostic models, clinical risk factors, and combination models, and may facilitate personalized risk stratification and treatment strategies for patients with nasopharyngeal carcinoma. To expand the generalizability of this prognostic model, validation with data from prospective, large sample, multicenter studies is required.

## Data Availability Statement

The original contributions presented in the study are included in the article/**Supplementary Material**. Further inquiries can be directed to the corresponding author.

## Ethics Statement

The studies involving human participants were reviewed and approved by Medical Ethics Committee of Sun Yat-sen University Cancer center. The patients/participants provided their written informed consent to participate in this study. Written informed consent was obtained from the individual(s) for the publication of any potentially identifiable images or data included in this article.

## Author Contributions

H-JL, L-ZL, YH, and G-YZ designed the research. H-JL and G-YZ performed the data analysis and drafted the manuscript. Y-BJ, X-PC, WL, J-CS, KC, and JZ provided scientific and technical support. H-JL, L-ZL and G-YZ collected clinical data. H-JL, YH, and G-YZ critically revised the manuscript. All authors critically reviewed the article and approved the final manuscript.

## Funding

This work was supported by the National Natural Science Foundation of China (No. 81572652), Science and Technology Planning Program of Guangdong Province (No. 2017A020215128), Science and Technology Planning Project of Guangzhou City (No. 201907010043), Foshan city climbing peak plan (No. 2019A009), and Science and Technology Innovation Platform of Foshan City (No. FS0AA-KJ218-1301-0007). The funding organizations had no role in the design and conduct of the study; collection, management, analysis, and interpretation of the data; preparation, review, or approval of the manuscript; and decision to submit the manuscript for publication.

## Conflict of Interest

The authors declare that the research was conducted in the absence of any commercial or financial relationships that could be construed as a potential conflict of interest.

## Publisher’s Note

All claims expressed in this article are solely those of the authors and do not necessarily represent those of their affiliated organizations, or those of the publisher, the editors and the reviewers. Any product that may be evaluated in this article, or claim that may be made by its manufacturer, is not guaranteed or endorsed by the publisher.

## Abbreviations

CI confidence interval; C-index concordance index; DMFS distant metastasis-free survival; EBV Epstein-Barr virus; NPC nasopharyngeal carcinoma; T1C-w contrast-enhanced; T1-weighted; T1-w T1-weighted; T2-w T2-weighted.
