# Supplementary material for: Establishment and Validation of a Novel MRI Radiomics Feature-Based Prognostic Model to Predict Distant Metastasis in Endemic Nasopharyngeal Carcinoma
Source: Front Oncol. 2022 Mar 23;12:794975. doi: 10.3389/fonc.2022.794975 (PMC8983880; doi:10.3389/fonc.2022.794975)
Supplement: Supplementary file 1 [file DataSheet_1.doc]

**Supplementary Materials**

**1.The inclusion criteria and exclusion criteria of this study**

**2.Chemotherapy for NPC patients**

**3.IMRT for NPC patients**

**4.MRI scan**

**5.Radiomics feature extraction**

**6.The Radscore calculation formulas of T1, T2 and T1C Radiomics prognostic models**

**7.The explanation for the nomogram and decision curve analysis (DCA)**

**8.Supplementary tables**

**8.1 Table S1. Univariate analysis of clinical characteristics for the primary cohort**

**8.2 Table S2. Multivariable analysis of clinical characteristics for the primary cohort**

**8.3 Table S3. Results of univariate analysis for radiomic features on the primary cohort**

**8.4 Table S4. The *P* value for C-index comparison between different prognostic models for DMFS prediction in the primary cohort and validation cohort**

**9.Supplementary figures**

**9.1 Figure S1. The process of Radiomics feature selection.** T1-w = T1-weighted; T2-w = T2-weighted; T1C-w = contrast-enhanced T1-weighted. ICC: Inter-class correlation coefficient; PCC = Pearson correlation coefficient; LASSO: Least absolute shrinkage and selection operator.

**9.2 Figure S2. Radiomics feature selection using the least absolute shrinkage and selection operator (LASSO) logistic regression model:** (A) T1-weighted Radiomics feature selection, (B) T2-weighted Radiomics feature selection, (C) contrast-enhanced T1-weighted Radiomics feature selection. Selection of tuning parameter (λ) in the LASSO model used 10-fold cross-validation via minimum criteria. The "deviance" curve which means partial-likelihood for the Cox model, was plotted versus log (λ). Dotted vertical lines were drawn at the optimal values by using the minimum criteria and the 1 standard error of the minimum criteria (the 1 - standard error criteria). The optimal λ value of 0.016 (T1), 0.030 (T2), or 0.012 (T1C) with log (λ) of -4.145 (T1), -3.508 (T2), or -4.386 (T1C) was chosen. LASSO coefficient profiles of the 8 (T1), 7 (T2), or 12 (T1C) selected features. A vertical line was plotted at the optimal λ value, which resulted in three scores with nonzero coefficients.

**9.3 Figure S3. Flowchart of the establishment of the prognostic models.** T1-w = T1-weighted; T2-w = T2-weighted; T1C-w = contrast-enhanced T1-weighted; T: tumor; N: node; EBV DNA: Plasma Epstein-Barr Virus DNA. MT1, MT2, MT1C, MT1+T2, MT1+T1C, MT2+T1C, and MT1+T2+T1C prognostic models were built based on clinical risk factors (T stage, N stage, EBV-DNA) integrated with T1, T2, T1C, T1+T2, T1+T1C, T2+T1C, and T1+T2+T1C radiomics prognostic models, respectively.

**9.4 Figure S4. Optimal risk score cutoff value selection using maximally selected rank statistic.** A) in the primary cohort of the clinical prognostic model, B) in the primary cohort of T1+T1C prognostic model.

**9.5 Figure S5. Kaplan-Meier survival curves of distant metastasis-free survival (DMFS) for patients in the low- and high-risk groups:** A) in the primary cohort of the clinical prognostic model, B) in the validation cohort of the clinical prognostic model, C) in the primary cohort of T1+T1C prognostic model, D) in the validation cohort of T1+T1C prognostic model.

**1.The inclusion criteria and exclusion criteria of this study**

The inclusion criteria of this study were as followings: (1) pathological confirmation of NPC by biopsy; (2) age ≥ 18 years and ≤ 70 years; (3) ECOG of 0 to 2; (4) received radical intensity modulated radiotherapy (IMRT); and (5) complete clinical data including plasma Epstein-Barr virus [EBV] DNA load; (6)adequate hematologic, renal, and hepatic function. The exclusion criteria of this study were as followings: (1) history of previous anticancer therapy (n=9); (2) primary malignant tumors in any other areas (n=6); (3) pregnancy and lactation (n=5); (4) The presence of distant metastasis before treatment (n=43); (5) inadequate hematologic, renal, and hepatic function (n=12); (6) failed to complete radiation therapy (n=5); (7) deviated from institutional guidelines due to organ dysfunction (n=13); (8) lack of follow-up or incomplete clinical data (n=32).

**2.Chemotherapy for NPC patients**

Concurrent chemotherapy consisted of 30-40 mg/m^2^ cisplatin weekly, or 80-100 mg/m^2^ cisplatin on day 1, 22 and 43 of radiotherapy. Induction chemotherapy consisted of docetaxel (60 mg/m^2^ on day 1), cisplatin (60 mg/m^2^ on day 1) and fluorouraci(600 mg/m^2^ per day from day 1 to day 5), or gemcitabine (1000 mg/m^2^ on day 1 and day 8) and cisplatin (80 mg/m^2^ on day 1), repeated every 21 days for 2 or 3 cycles.

**3.IMRT for NPC patients**

Target volumes were delineated slice-by-slice on the treatment planning CT scans using an individualized delineation protocol in accordance with International Commission on Radiation Units and Measurements reports 62 and 83. The gross tumor volumes (GTVs), including the primary tumor (GTVnx) and involved cervical [lymph node](app:ds:cervical lymph node)s (GTVnd), was determined from the imaging and endoscopic findings at presentation. The clinical target volume for the high-risk regions (CTV-1) included the GTVnx with a 5-10 mm margin (if possible) and the whole nasopharynx. The CTV for the low-risk regions (CTV-2) covered the potential sites of local infiltration (including the parapharyngeal space, posterior third of the nasal cavities and maxillary sinuses, pterygoid processes, lower one half of the sphenoid sinus, anterior one half of the clivus, and petrous tips) and bilateral cervical lymphatics down to the supraclavicular fossa. The planning target volumes (PTVs) were determined from the GTVs or CTVs, with a 3-5 mm margin for setup variations. The radiation doses for each target volume were as follows: 68-70 Gy in 30-33 fractions to the PTV of GTVnx; 60–68 Gy to the PTV of GTVnd; 60-62 Gy to the PTV of CTV-1; and 54 Gy to the PTV of CTV-2. According to our institutional treatment protocol, visible retropharyngeal [lymph node](app:ds:cervical lymph node)s in the MRI scans were incorporated into the primary tumor when delineating target volume, and received the same dose (68-70 Gy). The goal was to deliver ≥ 95% of the prescribed dose to 100% of the target volume. Details of the IMRT techniques used at the SYSUCC were also described in previous studies. (*1. Zhao C, Han F, Lu LX, et al. Intensity modulated radiotherapy for local-regional advanced nasopharyngeal carcinoma. Ai Zheng. 2004;23(11 Suppl):1532-1537; 2. Ma J, Liu L, Tang L, et al. Retropharyngeal lymph node metastasis in nasopharyngeal carcinoma: prognostic value and staging categories. Clin Cancer Res. 2007;13(5):1445-52.*)

**4.MRI scan**

T1-weighted images (TI-w) in the axial, coronal and sagittal planes (repetition time: 540 ms; echo time: 1.8 ms), and T2-weighted (T2-w) images in the axial plane (repetition time: 4000 ms; echo time: 99 ms) were acquired before intravenously administering gadolinium-based contrast material at a dose of 0.1 mmol/kg body weight. Following intravenous injection, contrast-enhanced T1-weighted (T1C-w) axial and sagittal sequences, and T1C-w fat-suppressed coronal sequences were performed sequentially, with parameters similar to those applied before the contrast material injection. The section thicknesses were 5 mm, 4 mm and 3 mm for the axial, sagittal and coronal planes, respectively, with a 1 mm interslice gap.

1. **Radiomics feature extraction**

In this study, a total of 4527 candidate radiomics features were generated from the delineated tumor region of axial T1-w, T2-w and T1C-w axial images (1509 features from each unimodal MR images). The 4527 features were divided into four types: first-order statistics features, shape (2D, 3D) features, textural features, and features after wavelet transform.

**First order statistics features:** First-order statistics describe the distribution of voxel intensities within the MRI image through commonly used and basic metrics. Nineteen first-order statistics features were used, including Energy, Total Energy, Entropy, Minimum,10th percentile, 90th percentile, Maximum, Mean, Median, Interquartile Range, Range, Mean Absolute Deviation (MAD), Robust Mean Absolute Deviation (rMAD), Root Mean Squared (RMS), Standard Deviation, Skewness,  Kurtosis, Variance, and Uniformity.

**Shape (2D, 3D) features:** In this group of features we included descriptors of the two- or three-dimensional size and shape of the ROI. These features are independent from the gray level intensity distribution in the ROI and are therefore only calculated on the non-derived image and mask. Ten two-dimensional size and shape based features were used, including Mesh Surface, Pixel Surface, Perimeter, Perimeter to Surface ratio, Sphericity, Spherical Disproportion, Maximum 2D diameter, Major Axis Length, Minor Axis Length, and Elongation. Seventeen three-dimensional size and shape based features were used, including Mesh Volume, Voxel Volume, Surface Area, Surface Area to Volume ratio, Sphericity, Compactness 1, Compactness 2, Spherical Disproportion, Maximum 3D diameter, Maximum 2D diameter (Slice), Maximum 2D diameter (Column), Maximum 2D diameter (Row), Major Axis Length, Minor Axis Length, Least Axis Length,  Elongation, and Flatness.

**Textural features:** Textural features are visual characteristics that reflect the homogeneity phenomenon of images and the arrangement of properties that change slowly or periodically on the body surface. Our textural features mainly included four typical matrices: the Gray Level Co-occurrence Matrix (GLCM), the Gray Level Run Length Matrix (GLRLM), the Gray Level Size Zone Matrix (GLSZM), the Neighbouring Gray Tone Difference Matrix (NGTDM), and the Gray Level Dependence Matrix (GLDM). We extracted 24 radiomics features from the GLCM, 16 features from the GLRLM, 16 features from the GLSZM, 5 radiomics features from the NGTDM, and 14 features from the GLDM, respectively.

The radiomics features in the GLCM mainly consisted of Autocorrelation, Joint Average, Cluster Prominence, Cluster Shade,  Cluster Tendency, Contrast, Correlation, Difference Average, Difference Entropy, Difference Variance, Joint Energy, Joint Entropy, Informational Measure of Correlation (IMC) 1,  Informational Measure of Correlation (IMC) 2, Inverse Difference Moment (IDM), Maximal Correlation Coefficient (MCC), Inverse Difference Moment Normalized (IDMN), Inverse Difference (ID), Inverse Difference Normalized (IDN), Inverse Variance, Maximum Probability, Sum Average, Sum Entropy, and Sum of Squares.

The radiomics features in the GLRLM mainly consisted of Short Run Emphasis (SRE), Long Run Emphasis (LRE), Gray Level Non-Uniformity (GLN), Gray Level Non-Uniformity Normalized (GLNN), Run Length Non-Uniformity (RLN), Run Length Non-Uniformity Normalized (RLNN), Run Percentage (RP), Gray Level Variance (GLV), Run Variance (RV), Run Entropy (RE), Low Gray Level Run Emphasis (LGLRE), High Gray Level Run Emphasis (HGLRE), Short Run Low Gray Level Emphasis (SRLGLE), Short Run High Gray Level Emphasis (SRHGLE), Long Run Low Gray Level Emphasis (LRLGLE), and  Long Run High Gray Level Emphasis (LRHGLE).

The radiomics features in the GLSZM mainly consisted of Small Area Emphasis (SAE), Large Area Emphasis (LAE), Gray Level Non-Uniformity (GLN), Gray Level Non-Uniformity Normalized (GLNN), Size-Zone Non-Uniformity (SZN), Size-Zone Non-Uniformity Normalized (SZNN), Zone Percentage (ZP), Gray Level Variance (GLV),  Zone Variance (ZV), Zone Entropy (ZE), Low Gray Level Zone Emphasis (LGLZE), High Gray Level Zone Emphasis (HGLZE), Small Area Low Gray Level Emphasis (SALGLE), Small Area High Gray Level Emphasis (SAHGLE), Large Area Low Gray Level Emphasis (LALGLE), and Large Area High Gray Level Emphasis (LAHGLE).

The radiomics features in the NGTDM mainly consisted of Coarseness, Contrast, Busyness, Complexity, and Strength.

The radiomics features in the GLDM mainly consisted of Small Dependence Emphasis (SDE), Large Dependence Emphasis (LDE), Gray Level Non-Uniformity (GLN), Dependence Non-Uniformity (DN), Dependence Non-Uniformity Normalized (DNN), Gray Level Variance (GLV), Dependence Variance (DV), Dependence Entropy (DE), Low Gray Level Emphasis (LGLE), High Gray Level Emphasis (HGLE), Small Dependence Low Gray Level Emphasis (SDLGLE), Small Dependence High Gray Level Emphasis (SDHGLE), Large Dependence Low Gray Level Emphasis (LDLGLE), and Large Dependence High Gray Level Emphasis (LDHGLE).

**Wavelet features:** The undecimated three-dimensional (3D) wavelet transform was used to decompose the original image, which can be regarded as a preprocessing prior to feature extraction. By changing the ratio of high-frequency to low-frequency signal in images, wavelet transform increases the information of low-frequency signal. Consider L and H to be a low-pass and high-pass functions respectively, X to be the decomposing image, and the wavelet decompositions of X to be labeled as
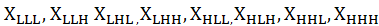
. Then, we can obtain eight new images which are decomposed in three directions (x, y, z). The size of each decomposition is equal to the original image and each decomposition is shift invariant. For each decomposition, we computed the first-order statistics and textural features described above.

Detailed information of radiomics features is presented on website (http://www.radiomics.io/pyradiomics.html).

**6.The Radscore calculation formulas of T1, T2 and T1C Radiomics prognostic models**

Rad-score (T1-w) = 0.01*T1_shape_Sphericity

+ 0.23*T1_WLHH_GLSZM_LGLZE

+ 1.78E-3*T1_WHHL_GLCM_IMC2

+138.93*T1_WHHH_GLCM_IMC2

+ 0.03*T1_WHLH_GLCM_IMC2

+ 3.91*T1_log.sigma.3.0.mm.3D_NGTDM_Strength

+ 4.37e-07*T1_WHHH_NGTDM_Contrast

Rad-score (T2-w) = 1.00*T2_WHLL_GLDM_LDHGLE

+ 0.64*T2_log.sigma.5.0.mm.3D_FOS_Skewness

+ 6.25E-11*T2_WHHL_GLSZM_SALGLE

+ 4.48E+07*T2_logarithm_NGTDM_Coarseness

+ 8.86E+10*T2_WLLH_GLCM_IDMN

Rad-score (T1C-w) = 7.30E+02*T1C_WHLL_GLCM_Correlation

+ 1.03*T1C_WLLH_GLSZM_SAHGLE

+ 1.14E+04*T1C_Gradient_GLCM_IMC1

+ 25.02*T1C_Square_GLCM_Correlation

+ 1.83*T1C_Gradient_GLSZM_ZE

+ 0.19*T1C_square_GLRLM_RE

+ 2.55*T1C_log.sigma.3.0.mm.3D_GLSZM_ZE

+ 0.35*T1C_WLHL_GLSZM_ZE

**+ 1.04*T1C_gradient_GLSZM_GLN**

| Variables | Range | | HR (95% CI) | P value |
| --- | --- | --- | --- | --- |
|  | Min~max | Median (IQR) |  |  |
| T1 radscore | -3.884~2.083 | 0.138(-0.546,0.660) | 2.72(1.93,3.82) | <.001 |
| T2 radscore | -3.800~2.695 | 0.087(-0.41,0.612) | 2.72(2.01,3.67) | <.001 |
| T1C radscore | -7.385~2.738 | 0.097(-0.492,0.697) | 2.72(2.04,3.63) | <.001 |

***Note*:** Min: minimum; max: maximum; IQR: inter-quartile range; HR: hazard ratio; CI: confidence interval.

**7.The explanation for the nomogram and decision curve analysis (DCA)**

**The explanation for the nomogram**

Radscore from nomogram. Nomogram function is from R’s package {rms}. Draws a partial nomogram that can be used to manually obtain predicted values from a regression model that was fitted with rms. The nomogram does not have lines representing sums, but it has a reference line for reading scoring points (default range 0–100). Once the reader manually totals the points, the predicted values (risk) can be read at the bottom.

**The explanation for the Decision curve analysis (DCA)**

Decision curve analysis (DCA) are performed on the R’s packages “dca.R”. DCA was conducted to determine the clinical usefulness of the nomogram by calculating the net benefits at varying values of threshold probability in the training and validation datasets. ^1^ Net benefit yields the proportion of “net” true positives in the dataset: the observed number of true positives is corrected for the observed proportion of false positives weighted by the odds of the risk threshold, and the result is divided by the sample size.^2^ The x-axis represents the threshold probability, and the Y-axis shows the net benefit.

*References*

1. *Zhang Z, Rousson V, Lee WC, Ferdynus C, Chen M, Qian X, et al. Decision curve analysis: a technical note. Ann Transl Med 2018;6(15):308. https://doi.org/10.21037/atm.2018.07.02.*
2. *Van Calster B, Wynants L, Verbeek JFM, Verbakel JY, Christodoulou E, Vickers AJ, et al. Reporting and Interpreting Decision Curve Analysis: A Guide for Investigators. Eur Urol 2018; 74(6): 796–804. https://doi.org/10.1016/j.eururo.2018.08.038.*

**Table S1. Un**ivariate analysis of clinical characteristics for the primary cohort

| Variable | Numbers^#^ | DMFS | | |
| --- | --- | --- | --- | --- |
|  |  | 5-year DMFS | HR (95% CI) | *P* |
| Age (years) |  |  |  | 0.067 |
|  | 44(38~53) | - | 1.02 (1.00, 1.04) |  |
| Gender |  |  |  |  |
| Male | 371 (71.6%) | 88.12% | Reference | Reference |
| Female | 147 (28.4%) | 90.30% | 0.90 (0.52, 1.54) | 0.699 |
| WHO pathologic classification |  |  |  |  |
| Type I/II | 27 ( 5.2%) | 77.19% | Reference | Reference |
| Type III | 491(94.8%) | 89.37% | 0.50 (0.22, 1.16) | 0.106 |
| EBV DNA (10^3^ copies/ml)* |  |  |  |  |
| < 1 | 232 (44.8%) | 95.55% | Reference | Reference |
| < 10 | 117 (22.6%) | 84.07% | 3.67 (1.85, 7.28) | 0.002 |
| < 100 | 129 (24.9%) | 82.74% | 3.72 (1.89, 7.30) | <0.001 |
| ≥ 100 | 40 ( 7.7%) | 81.91% | 3.92 (1.62, 9.46) | <0.001 |
| T classification |  |  |  |  |
| T1 | 140 (27.0%) | 95.64% | Reference | Reference |
| T2 | 60 (11.6%) | 89.60% | 2.59 (1.11, 6.03) | 0.028 |
| T3 | 186 (35.9%) | 86.60% | 3.32 (1.23, 8.91) | 0.017 |
| T4 | 132 (25.5%) | 80.89% | 4.85 (2.12, 11.12) | <0.001 |
| N classification |  |  |  |  |
| N0 | 128 (24.7%) | 96.07% | Reference | Reference |
| N1 | 282 (54.4%) | 90.56% | 2.46 (1.03, 5.91) | 0.043 |
| N2 | 69 (13.3%) | 80.35% | 6.84 (2.48, 18.83) | <0.001 |
| N3 | 39 ( 7.5%) | 71.60% | 7.80 (3.13, 19.45) | <0.001 |
| AJCC clinical stage (2010) |  |  |  |  |
| I | 53 (10.2%) | 98.11% | Reference | Reference |
| II | 117 (22.6%) | 94.02% | 3.73 (0.47, 29.84) | 0.214 |
| III | 185 (35.7%) | 88.45% | 7.07 (0.95, 52.33) | 0.056 |
| IVa | 163 (31.5%) | 81.78% | 13.46 (1.84, 98.24) | 0.010 |
| Treatment regimen |  |  |  |  |
| RT alone | 71 (13.7%) | 95.75% | Reference | Reference |
| CCRT | 201 (38.8%) | 87.85% | 2.66 (0.93, 7.6) | 0.067 |
| IC + CCRT | 246 (47.5%) | 87.38% | 2.74 (0.98, 7.72) | 0.056 |
| Blood type |  |  |  |  |
| A | 139 (26.8%) | 86.51% | Reference | Reference |
| B | 130 (25.1%) | 91.42% | 0.64 (0.32, 1.27) | 0.201 |
| AB | 17 ( 3.3%) | 94.12% | 0.77 (0.18, 3.28) | 0.722 |
| o | 232 (44.8%) | 88.15% | 0.86 (0.49, 1.49) | 0.580 |
| HBsAg |  |  |  |  |
| Negative | 427 (82.4%) | 89.43% | Reference | Reference |
| Positive | 91 (17.6%) | 85.52% | 1.25 (0.69, 2.24) | 0.465 |
| LDH (U/L)* |  |  |  |  |
| < 245 | 492 (95%) | 88.77% | Reference | Reference |
| ≥ 245 | 26 ( 5%) | 88.11% | 0.97 (0.3, 3.08) | 0.956 |
| hs-CRP (g/ml)* |  |  |  |  |
| < 1 | 196 (37.8%) | 88.6% | Reference | Reference |
| 1-3 | 166 (32.0%) | 91.38% | 0.79 (0.42, 1.49) | 0.475 |
| ≥ 3 | 156 (30.1%) | 86.06% | 1.46 (0.84, 2.52) | 0.181 |
| Platelet counts (10^9^/L)* |  |  |  |  |
| < 100 | 4 ( 0.8%) | 75.00% | Reference | Reference |
| 100-300 | 436 (84.2%) | 88.52% | 0.49 (0.07, 3.53) | 0.477 |
| ≥ 300 | 78 (15.1%) | 90.62% | 0.43 (0.05, 3.4) | 0.425 |
| Leucocyte counts (10^9^/L)* |  |  |  |  |
| < 10 | 466 ( 90.0%) | 89.50% | Reference | Reference |
| ≥ 10 | 52 (10.0%) | 81.25% | 1.57 (0.78, 3.17) | 0.204 |

***Note*:** Hazard ratios estimated by Cox proportional hazards regression. All statistical tests were two-sided. DMFS: distant metastasis free survival; HR: hazard ratio; CI: confidence interval; WHO: World Health Organization; Type I: keratinizing; Type II: non-keratinizing differentiated; Type III: non-keratinizing undifferentiated; EBV DNA: Plasma Epstein-Barr Virus DNA; T: tumor; N: node; AJCC: American Joint Committee on Cancer; RT: radiotherapy; CCRT: concurrent chemoradiothrapy; IC: induction chemotherapy; HBsAg: hepatitis B surface antigen; LDH: serum lactate dehydrogenase levels; hs-CRP: high-sensitivity C-reactive protein.

* The result before treatment.

^#^ In the parentheses are Percentages.

**Table S2.** Multivariable analysis of clinical characteristics for the primary cohort

| Prognostic model | Variable | DMFS | | |
| --- | --- | --- | --- | --- |
|  |  | coefficient | HR (95% CI) | *P* |
| Clinical prognosticmodel | EBV DNA (10^3^ copies/ml)* |  |  |  |
|  | < 1 | Reference | | |
|  | < 10 | 0.56 | 1.74 (0.68, 4.48) | 0.249 |
|  | < 100 | 0.63 | 1.88 (0.9, 3.91) | 0.091 |
|  | ≥ 100 | 0.84 | 2.31 (1.13, 4.75) | 0.022 |
|  | T classification |  |  |  |
|  | T1 | Reference | | |
|  | T2 | 0.59 | 1.81 (0.77, 4.26) | 0.174 |
|  | T3 | 0.83 | 2.29 (0.84, 6.21) | 0.105 |
|  | T4 | 1.21 | 3.35 (1.45, 7.77) | 0.005 |
|  | N classification |  |  |  |
|  | N0 | Reference | | |
|  | N1 | 0.59 | 1.81 (0.74, 4.41) | 0.192 |
|  | N2 | 1.40 | 4.06 (1.38, 11.93) | 0.011 |
|  | N3 | 1.47 | 4.37 (1.65, 11.55) | 0.003 |

***Note*:** Hazard ratios estimated by Cox proportional hazards regression. All statistical tests were two-sided. DMFS: distant metastasis free survival; HR: hazard ratio; CI: confidence interval; EBV DNA: Plasma Epstein-Barr Virus DNA; T: tumor; N: node.

| MR image modality | Variables | Range | | HR (95% CI) | *P value* |
| --- | --- | --- | --- | --- | --- |
|  |  | Min~max | Median (IQR) |  |  |
| T1-W | T1_shape_Sphericity | 0.428~0.842 | 0.595 (0.553, 0.636) | 0.01 (2.70E-04, 0.49) | 0.019 |
|  | T1_WLHH_GLSZM_LGLZE | 0.051~0.672 | 0.225 (0.174, 0.417) | 0.13 (0.02, 0.78) | 0.026 |
|  | T1_WHHL_GLCM_IMC2 | 0.316~0.987 | 0.600 (0.507, 0.729) | 0.02 (3.24E-03, 0.14) | <0.001 |
|  | T1_WHHH_GLCM_IMC2 | 0.139~0.921 | 0.310 (0.239, 0.406) | 0.09 (0.01, 0.82) | 0.033 |
|  | T1_WHLH_GLCM_IMC2 | 0.145~0.930 | 0.312 (0.246, 0.414) | 0.02 (2.03E-03, 0.23) | 0.002 |
|  | T1_log.sigma.3.0.mm.3D_NGTDM_Strength | 0.017~2.629 | 0.215 (0.123, 0.406) | 0.35 (0.13, 0.99) | 0.048 |
|  | T1_WHHH_NGTDM_Contrast | 0.005~0.329 | 0.026 (0.018, 0.036) | 1.51E-16 (4.98E-26, 4.59E-07) | 0.001 |
| T2-W | T2_WHLL_GLDM_LDHGLE | 51.250~19050.207 | 1.51E3 (7.13E2, 2.92E3) | 1.000164 (1.000109, 1.000219) | <0.001 |
|  | T2_log.sigma.5.0.mm.3D_FOS_Skewness | -2.164~1.199 | 0.015 (-0.253, 0.249) | 0.41 (0.26, 0.65) | <0.001 |
|  | T2_WHHL_GLSZM_SALGLE | 0.004~0.193 | 0.036 (0.022, 0.056) | 1.83E-12 (3.36E-18, 1.00E-6) | <0.001 |
|  | T2_logarithm_NGTDM_Coarseness | 0.001~0.236 | 0.019 (0.009, 0.037) | 6.75E-06 (5.95E-11, 0.77) | 0.045 |
|  | T2_WLLH_GLCM_IDMN | 0.856~0.994 | 0.973 (0.961, 0.981) | 4.28E15 (4.27E15, 4.30E24) | <0.001 |
| T1C-W | T1C_WHLL_GLCM_Correlation | -0.518~0.194 | 0.020 (-0.020, 0.048) | 236.82 (4.23, 13265.71) | 0.008 |
|  | T1C_WLLH_GLSZM_SAHGLE | 2.003~76.771 | 15.706 (10.445, 24.144) | 1.03 (1.01, 1.05) | <0.001 |
|  | T1C_Gradient_GLCM_IMC1 | -0.487~0.000 | -0.055 (-0.076, -0.041) | 4.60E5 (6.99, 3.03E8) | 0.017 |
|  | T1C_Square_GLCM_Correlation | -0.384~0.636 | 0.238 (0.144, 0.318) | 22.10 (3.99, 122.47) | <0.001 |
|  | T1C_Gradient_GLSZM_ZE | 0.000~4.022 | 2.419 (2.000, 2.82) | 2.13 (1.34, 3.40) | 0.001 |
|  | T1C_square_GLRLM_RE | 1.326~3.904 | 2.970 (2.689, 3.237) | 2.03 (1.09, 3.80) | 0.026 |
|  | T1C_log.sigma.3.0.mm.3D_GLSZM_ZE | 2.00~5.819 | 3.882 (3.388, 4.282) | 2.50 (1.64, 3.81) | <0.001 |
|  | T1C_WLHL_GLSZM_ZE | 2.646~6.175 | 4.952 (4.611, 5.235) | 2.09 (1.22, 3.57) | 0.007 |
|  | T1C_gradient_GLSZM_GLN | 1.00~35.92 | 5.000 (3.222, 8.575) | 1.06 (1.03, 1.10) | <0.001 |

**Table S3. Results of univariate analysis for radiomic features on the primary cohort**

***Note*:** Min: minimum; max: maximum; IQR: inter-quartile range; HR: hazard ratio; CI: confidence interval; *p* value for each radiomic feature associated with outcome was calculated using Cox proportional hazards regression. Textural features should be decomposed into three dimensional wavelet transform (8 decompositions), and the wavelet decompositions are labeled as W_LLL_, W_LLH_, W_LHL_, W_LHH_, W_HLH_, W_HHL_, W_HHL_, and W_HHH_. T1-w = T1-weighted; T2-w = T2-weighted; T1C-w = contrast-enhanced T1-weighted. GLSZM: Gray Level Size Zone Matrix; GLCM: Gray Level Co-occurence Matrix; NGTDM: Neighbouring Gray Tone Difference Matrix; GLDM: Gray Level Dependence Matrix; FOS: First order statistics; GLRLM: Gray Level Run Length Matrix; LGLZE: Low Gray Level Zone Emphasis; IMC: Informational measure of correlation; JA: Joint Average; LDHGLE: Large Dependence High Gray Level Emphasis; SALGLE: Small Area Low Gray Level Emphasis; IDMN: Inverse Difference Moment Normalized; SAHGLE: Small Area High Gray Level Emphasis; ZE: Zone Entropy; RE: Run Entropy; GLN: Gray Level Non-Uniformity.

**Table S4. The *P* value for C-index comparison between different prognostic models for DMFS prediction in the primary cohort and validation co**hort

| Primary cohort | | | | | | | |
| --- | --- | --- | --- | --- | --- | --- | --- |
|  | T1 | T2 | T1C | T1+T2 | T1+T1C | T2+T1C | T1+T2+T1C |
| T1 | － | 0.554 | 0.347 | <0.001 | <0.001 | 0.012 | <0.001 |
| T2 | 0.554 | － | 0.215 | <0.001 | 0.002 | <0.001 | <0.001 |
| T1C | 0.347 | 0.215 | － | 0.165 | 0.001 | 0.002 | <0.001 |
| T1+T2 | <0.001 | <0.001 | 0.165 | － | 0.374 | 0.797 | 0.021 |
| T1+T1C | <0.001 | 0.002 | 0.001 | 0.374 | － | 0.922 | 0.082 |
| T2+T1C | 0.012 | <0.001 | 0.002 | 0.797 | 0.922 | － | 0.037 |
| T1+T2+T1C | <0.001 | <0.001 | <0.001 | 0.021 | 0.082 | 0.037 | － |
| Validation cohort | | | | | | | |
|  | T1 | T2 | T1C | T1+T2 | T1+T1C | T2+T1C | T1+T2+T1C |
| T1 | － | 0.621 | 0.254 | 0.189 | 0.008 | 0.240 | 0.025 |
| T2 | 0.621 | － | 0.135 | 0.015 | 0.043 | 0.003 | 0.001 |
| T1C | 0.254 | 0.135 | － | 0.616 | 0.048 | 0.355 | 0.161 |
| T1+T2 | 0.189 | 0.015 | 0.616 | － | 0.162 | 0.279 | 0.019 |
| T1+T1C | 0.008 | 0.043 | 0.048 | 0.162 | － | 0.466 | 0.825 |
| T2+T1C | 0.240 | 0.003 | 0.355 | 0.279 | 0.466 | － | 0.115 |
| T1+T2+T1C | 0.025 | 0.001 | 0.161 | 0.019 | 0.825 | 0.115 | － |
| Primary cohort | | | | | | | |
|  | MT1 | MT2 | MT1C | MT1+  T2 | MT1+  T1C | MT2+  T1C | MT1+T2+  T1C |
| MT1 | － | 0.563 | 0.637 | <0.001 | <0.001 | 0.015 | <0.001 |
| MT2 | 0.563 | － | 0.465 | 0.001 | 0.006 | 0.001 | <0.001 |
| MT1C | 0.637 | 0.465 | － | 0.090 | <0.001 | <0.001 | <0.001 |
| MT1+T2 | <0.001 | 0.001 | 0.090 | － | 0.256 | 0.725 | 0.006 |
| MT1+T1C | <0.001 | 0.006 | <0.001 | 0.256 | － | 0.960 | 0.020 |
| MT2+T1C | 0.015 | 0.001 | <0.001 | 0.725 | 0.960 | － | 0.013 |
| MT1+T2+T1C | <0.001 | <0.001 | <0.001 | 0.006 | 0.020 | 0.013 | － |
| Validation cohort | | | | | | | |
|  | MT1 | MT2 | MT1C | MT1+  T2 | MT1+  T1C | MT2+  T1C | MT1+T2+  T1C |
| MT1 | － | 0.460 | 0.608 | 0.321 | 0.004 | 0.396 | 0.038 |
| MT2 | 0.460 | － | 0.179 | 0.010 | 0.033 | 0.002 | <0.001 |
| MT1C | 0.608 | 0.179 | － | 0.948 | 0.016 | 0.258 | 0.008 |
| MT1+T2 | 0.321 | 0.010 | 0.948 | － | 0.194 | 0.547 | 0.010 |
| MT1+T1C | 0.004 | 0.033 | 0.016 | 0.194 | － | 0.352 | 0.720 |
| MT2+T1C | 0.396 | 0.002 | 0.258 | 0.547 | 0.352 | － | 0.045 |
| MT1+T2+T1C | 0.038 | <0.001 | 0.008 | 0.010 | 0.720 | 0.045 | － |

***Note*:** DMFS: distant metastasis free survival. MT1, MT2, MT1C, MT1+T2, MT1+T1C, MT2+T1C and MT1+T2+T1C prognostic models were built that integrated the clinical risk factors (T stage, N stage, and plasma EBV DNA) with the T1, T2, T1C, T1+T2, T1+T1C, T2+T1C and T1+T2+T1C radiomics prognostic model, respectively.


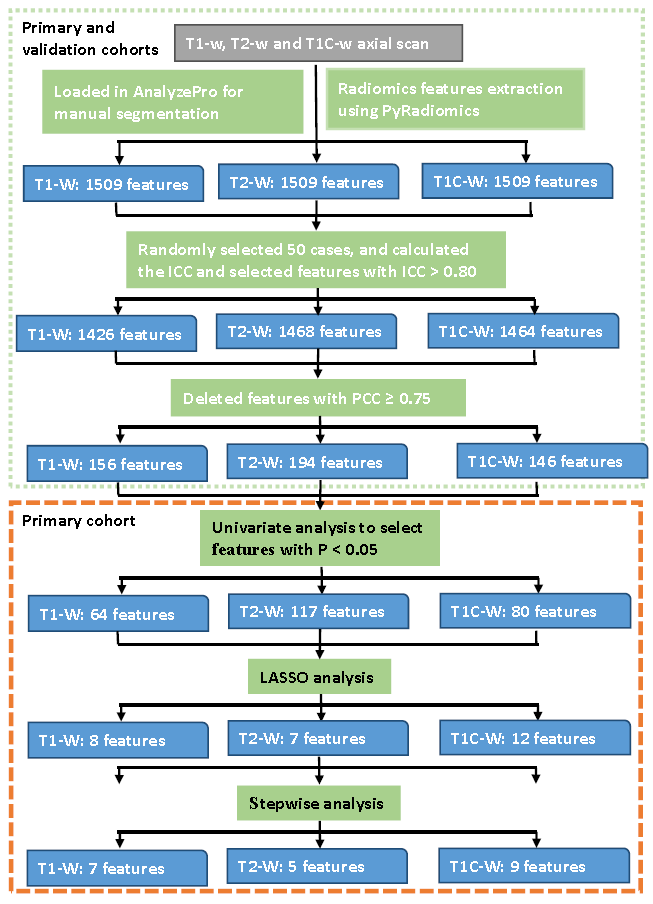


**Supplementary Figure S1. The process of Radiomics feature selection.** T1-w = T1-weighted; T2-w = T2-weighted; T1C-w = contrast-enhanced T1-weighted. ICC: Inter-class correlation coefficient; PCC = Pearson correlation coefficient; LASSO: Least absolute shrinkage and selection operator.


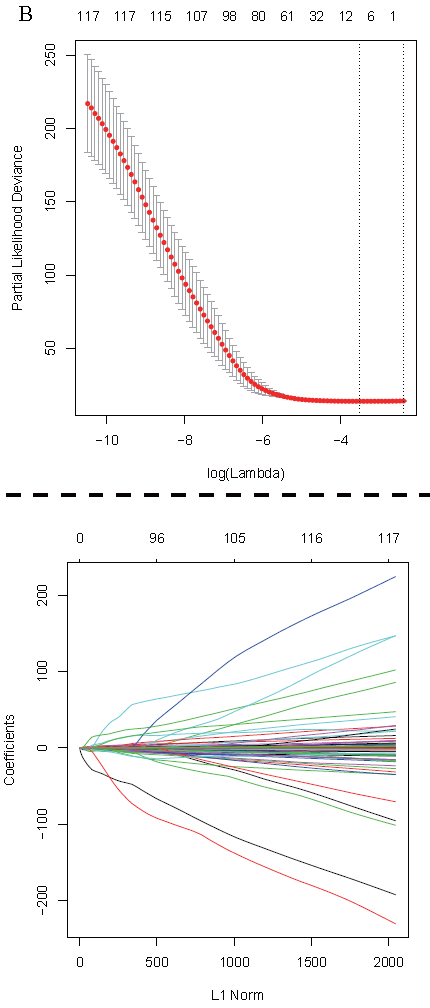

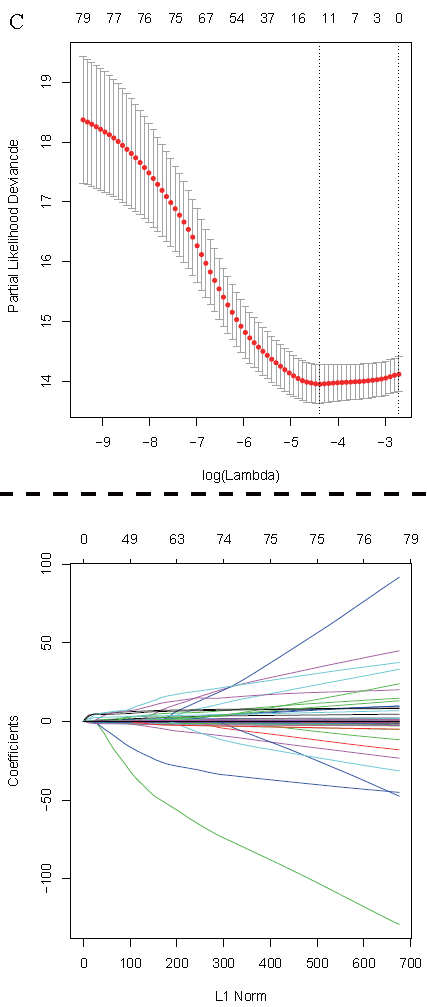

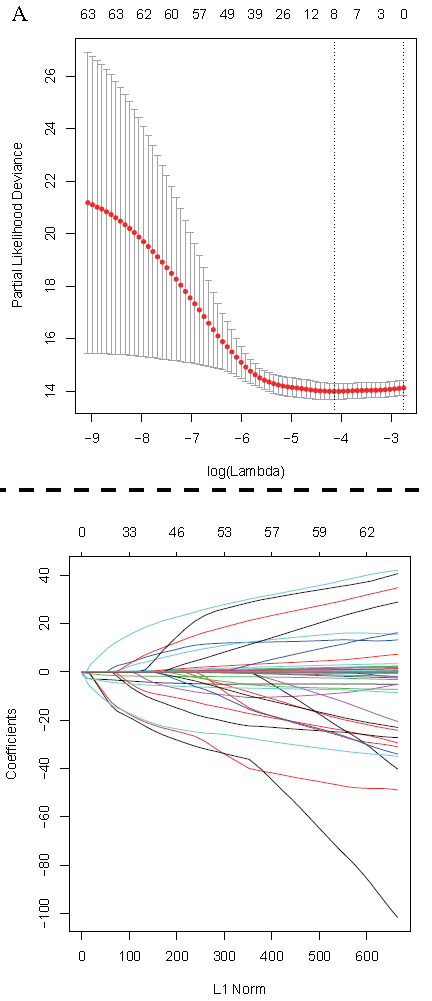


**Supplementary Figure S2. Radiomics feature selection using the least absolute shrinkage and selection operator (LASSO) logistic regression model:** (A) T1-weighted Radiomics feature selection, (B) T2-weighted Radiomics feature selection, (C) contrast-enhanced T1-weighted Radiomics feature selection. Selection of tuning parameter (λ) in the LASSO model used 10-fold cross-validation via minimum criteria. The "deviance" curve which means partial-likelihood for the Cox model, was plotted versus log (λ). Dotted vertical lines were drawn at the optimal values by using the minimum criteria and the 1 standard error of the minimum criteria (the 1 - standard error criteria). The optimal λ value of 0.016 (T1), 0.030 (T2), or 0.012 (T1C) with log (λ) of -4.145 (T1), -3.508 (T2), or -4.386 (T1C) was chosen. LASSO coefficient profiles of the 8 (T1), 7 (T2), or 12 (T1C) selected features. A vertical line was plotted at the optimal λ value, which resulted in three scores with nonzero coefficients.


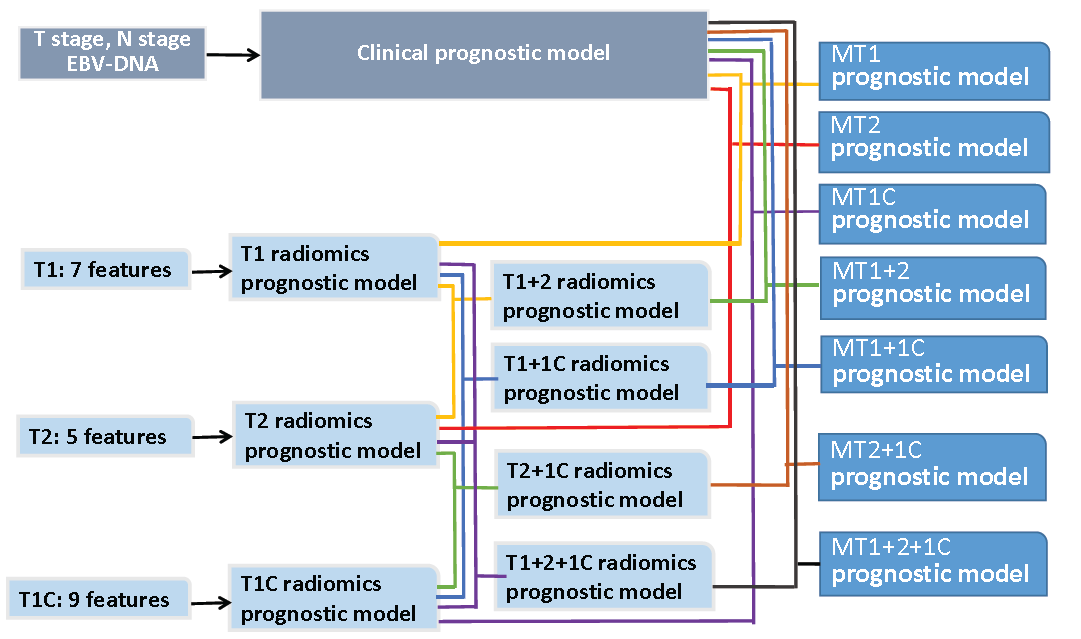


**Supplementary Figure S3. Flowchart of the establishment of the prognostic models.** T1-w = T1-weighted; T2-w = T2-weighted; T1C-w = contrast-enhanced T1-weighted; T: tumor; N: node; EBV DNA: Plasma Epstein-Barr Virus DNA. MT1, MT2, MT1C, MT1+T2, MT1+T1C, MT2+T1C, and MT1+T2+T1C prognostic models were built based on clinical risk factors (T stage, N stage, EBV-DNA) integrated with T1, T2, T1C, T1+T2, T1+T1C, T2+T1C, and T1+T2+T1C radiomics prognostic models, respectively.


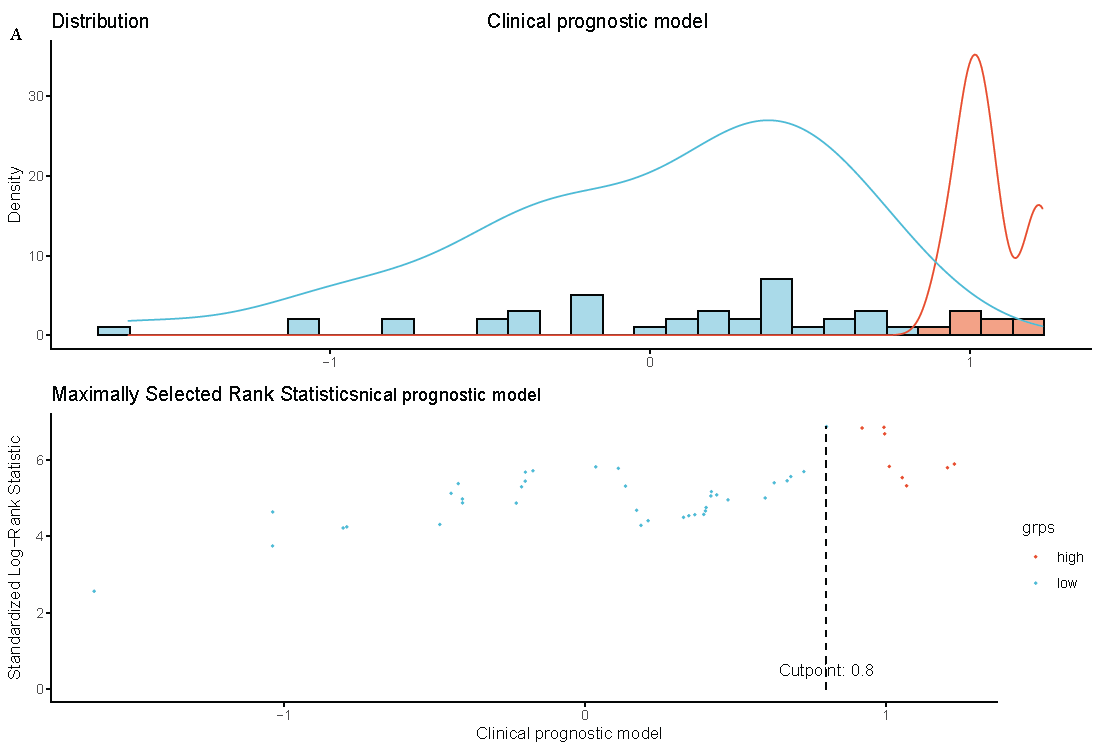


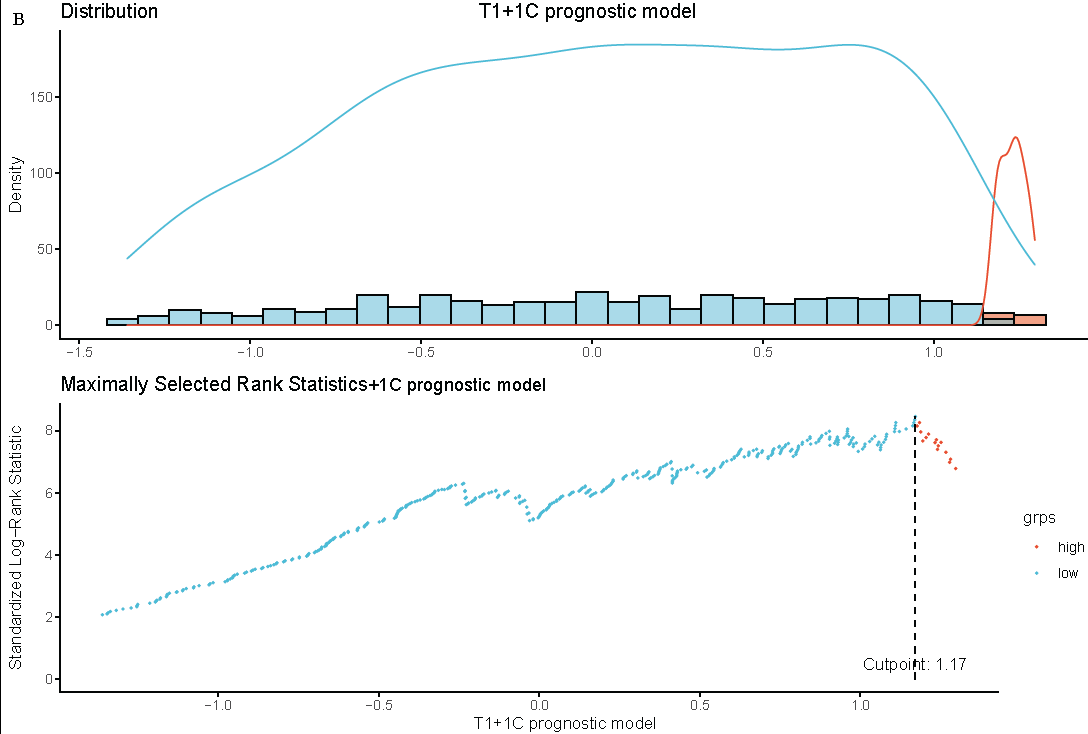


**Supplementary Figure S4. Optimal risk score cutoff value selection using maximally selected rank statistic. A**) in the primary cohort of the clinical prognostic model, B) in the primary cohort of T1+T1C prognostic model.


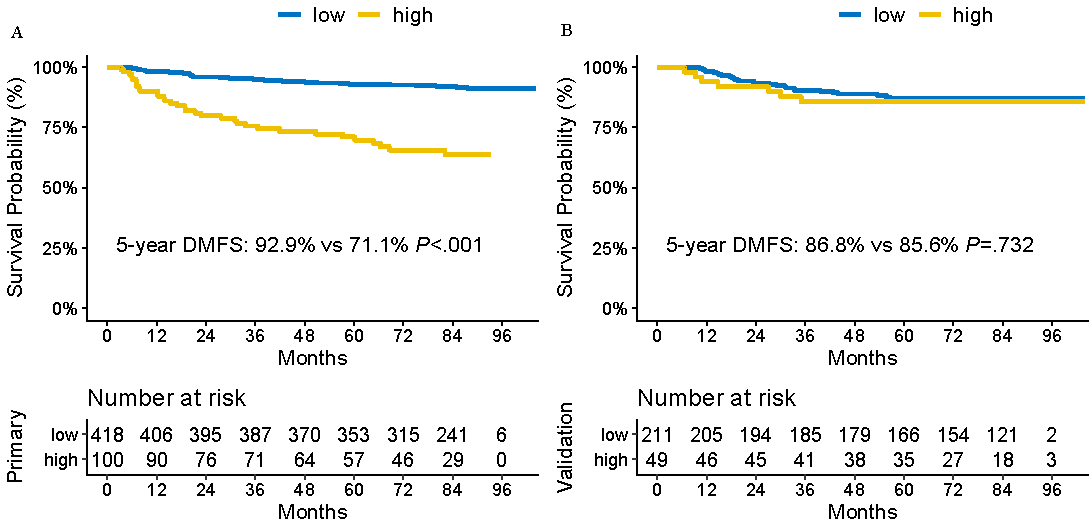


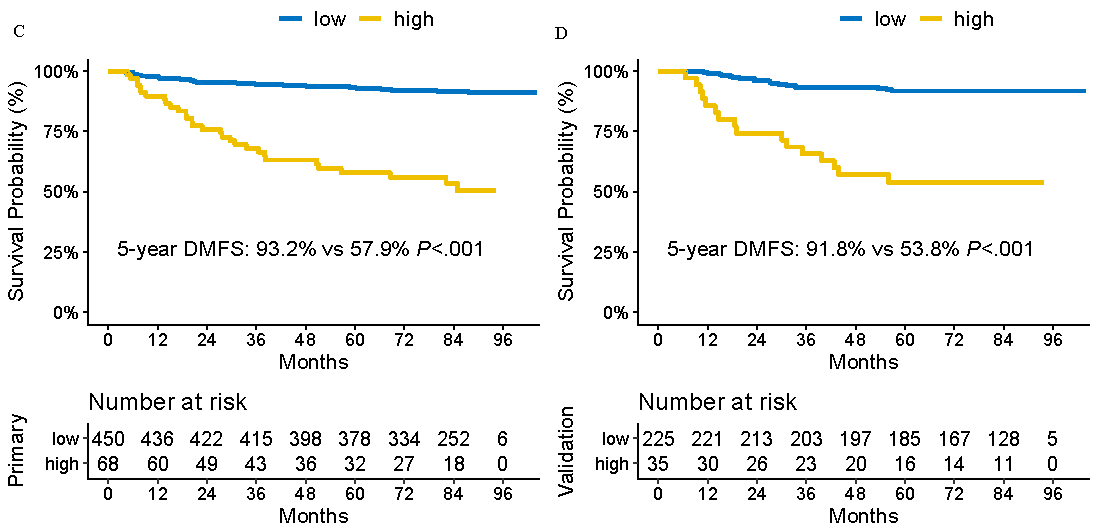


**Supplementary Figure S5. Kaplan-Meier survival curves of distant metastasis-free survival (DMFS) for patients in the low- and high-risk groups:** A) in the primary cohort of the clinical prognostic model, B) in the validation cohort of the clinical prognostic model, C) in the primary cohort of T1+T1C prognostic model, D) in the validation cohort of T1+T1C prognostic model.
